# Structural Equation Modeling of Drivers’ Situation Awareness Considering Road and Driver Factors

**DOI:** 10.3389/fpsyg.2020.01601

**Published:** 2020-07-21

**Authors:** Yanqun Yang, Meifeng Chen, Changxu Wu, Said M. Easa, Xinyi Zheng

**Affiliations:** ^1^College of Civil Engineering, Fuzhou University, Fuzhou, China; ^2^Department of Industrial Engineering, Tsinghua University, Beijing, China; ^3^Department of Civil Engineering, Ryerson University, Toronto, ON, Canada; ^4^Department of Humanities and Social Sciences, School of Humanities and Social Sciences, Fuzhou University, Fuzhou, China

**Keywords:** driver, situation awareness, influential factors, structural equation model, cognition

## Abstract

Driver’s situation awareness (SA) is one of the key elements that affect driving decision-making and driving behavior. SA is influenced by many factors, and previous studies have focused only on individual factors. This study presents a comprehensive study to explore the path relationships and influence mechanism between SA and all influential factors, including road characteristics, driver characteristics and states, distracting elements, and cognitive ability. A structural equation model that relates SA to its influential factors is developed. A total of 324 valid questionnaires were collected to analyze and identify the relationships between the factors. The results show that the preceding influential factors have significant effects on SA, which is consistent with previous research. Based on path coefficients, positive effects were: cognitive abilities (0.500), driver state (0.360), age (0.277), driving experience (0.198), and gender (0.156). Negative effects were: distracting elements (−0.253) and road characteristics (−0.213). The results of this comprehensive study provide a valuable reference for the development of driver training programs and driving regulations.

## Introduction

In the field of driving safety, Zheng (2018) reported that more than 80% of traffic accidents were due to drivers’ ability to navigate the roads. Drivers must constantly perceive and understand road traffic conditions, predict possible hazards, and quickly make and execute safe driving decisions. The dynamics and complexity of traffic environments place a high demand on driver’s situation awareness (SA). Young et al. (2013) proposed that driver’s SA specifically refers to driver understanding of the relationship among driving objectives (e.g., driving according to traffic signs, road conditions, and weather conditions), driving behavior of other drivers, and vehicle state. When SA level is too low to cope with current traffic situations, the risk of serious accidents increases. According to Endsley (1995a), the pioneer of SA, 88% of all aviation accidents caused by human error could be attributed to SA. Research has also shown that SA is a key factor in driver decision-making and behavior and the most common cause of errors in driving tasks (Gugerty, 1997; Durso et al., 2007). Road safety is clearly inextricably linked to SA.

The driver’s SA is influenced by many factors. While these factors have been widely studied, most analyses have only focused on one factor or a small group of factors from a quantitative perspective. A study inclusive of a comprehensive analysis of all factors (or even the main factors) that affect SA does not yet exist. SA and its influential factors are similar to many hypothetical constructs, such as attitudes, workload, and satisfaction. Although this presents the challenge of effectively measuring an abstract concept, quantitative data may be indirectly derived through multiple, measurable indicators.

The structural equation model (SEM) provides a means for measuring the relationship between multiple variables at the same time. SEM is mainly used to analyze and identify the relationship between multiple latent variables (Sadia et al., 2018). Therefore, the method can be used to discern the relationship between these factors and their influence on SA.

Based on the existing research on SA, this study used SEM to explore the relationship between influential factors of SA with the aim of providing suggestions to improve driver training and driving regulations. In our study, hypotheses for measurement and structural sub-models were established based on existing SEM literature. In the model hypothesis, a survey questionnaire was developed, and data were collected. Tests of validity and reliability, a model-fit test, and a hypothesis test were carried out using SPSS and AMOS software. Finally, the identified factors that affect SA and their interactions were analyzed using the load coefficient of the measurement sub-model and the path coefficient of the structural sub-model.

The next section presents the proposed methodology, including the factors that affect SA and the structural equation model. The following section presents the data collection process using questionnaires. The results of model validation and discussion are then presented, followed by the conclusions.

## Proposed Methodology

### Factors Affecting Situation Awareness

The first step in developing the structural equation model was to identify the factors that influence SA. Endsley (1995b) proposed that SA was the internal representation of an individual’s changing the external environment. As traffic environments are dynamic and complex, driver’s SA could be affected by many discrete factors. At present, the most influential factors relative to SA that have been comprehensively discussed from the perspective of individual drivers and their external environments include age, driving experience, emotional state, level of fatigue, cognitive ability, distracting elements, and road characteristics. Each factor is discussed in turn below.

As drivers age, their physical fitness, perception, and cognitive abilities will change. The influence of age on SA varies considerably in different age groups. More specifically, the variability is seen the most between youth, middle-aged, and elderly drivers. In several studies, SA of youth drivers was found to be less than that of middle-aged drivers (Bolstad, 2001; Lee et al., 2006), while the overall SA of elderly drivers was lower than that of the youth drivers (Bolstad, 2001; Kaber et al., 2012; Liu and Cian, 2014; Key et al., 2017).

Experienced drivers have better SA than novice drivers (Kass et al., 2007; Jackson et al., 2009; Soliman and Mathna, 2009; Underwood et al., 2013; Crundall, 2016), which is mainly due to their visual search and perception abilities that are gained from practice. Experienced drivers also have flexible visual search patterns and can quickly identify potential hazards. Novice drivers’ visual attention tends to only be concentrated on one specific aspect of driving at a time, and they usually cannot obtain dangerous information quickly and accurately.

Emotions can easily affect people’s perception and judgment, and so their impact can spill over into driver’s SA. For the purpose of this study, we treat emotions as falling into one of three domains: positive, negative, and normal emotional states. Negative emotions can reduce driver’s SA and contribute to poor driving performance (Jeon et al., 2014). When drivers are fatigued, their level of perception and concentration will decline. Fatigue has been shown to have a significant impact on the three levels of SA: perception, understanding, and prediction (Wijayanto et al., 2016).

Cognitive ability is the ability of individuals to acquire and process internal and external information. The aspects of cognitive ability that affect driver’s SA mainly include visual processing skills, working memory capacity, spatial perceptual ability, and time-sharing ability (O’Hare, 1997; Bolstad, 2001; Johannsdottir and Herdman, 2010), all of which help drivers to maintain a high level of SA. In particular, working memory capacity and visual processing skills were identified as the most crucial factors necessary to maintain high SA levels during hazardous driving conditions (Kaber et al., 2016).

Distraction entails a driver diverting his/her attention from driving tasks to something unrelated to driving (Krueger, 2011). Driver distraction is mainly caused by the use of mobile phones, talking, and looking at roadside billboards. Distraction can reduce SA by commandeering limited cognitive resources and affecting their decision-making abilities and vehicle operation (Gugerty et al., 2003; Ma and Kaber, 2005; Kass et al., 2007; Rogers et al., 2011; Heenan et al., 2014; Kaber et al., 2016). However, some researchers argue that specific driving situations dictate whether drivers engage in distracting behaviors (Schömig and Metz, 2013; Young et al., 2016). For instance, when faced with traffic conditions that were complex, Young et al. (2016) found that drivers tended to quickly cease distracting tasks to cope with those traffic conditions.

Traffic environments are comprised of road users, traffic facilities, surrounding buildings, and billboards. Drivers must allocate more cognitive and attention resources to deal with traffic information on the road in high-complexity situations (Bolstad, 2001; Stanton and Young, 2005; Hartono and Gozali, 2015). This level of demand on drivers can result in a decrease in their SA levels.

While existing research as to the role of gender in promoting SA is mixed, with some studies finding that gender did not have a significant impact on SA in various driving scenarios (Heenan et al., 2014), some studies have found that gender could result in significant differences in driving behavior (Machin and Sankey, 2008; Zhang et al., 2018; Wang et al., 2019). This factor requires further investigation.

In summary, the comprehensive factors that affect SA, considered in this study, include road characteristics, driver characteristics (age, gender, and driving experience) and driver states (emotion and fatigue), distracting elements, and cognitive abilities. The relationships between SA and its influential factors is established using a structural equation model.

### Structural Equation Model

SEM is a multivariate statistical method that integrates factor analysis and path analysis. It concretizes latent variables that are difficult to directly observe through several observed variables and establishes the relationship among those latent variables (Martynova et al., 2018). SEM is a verifiable analysis method, consisting of measurement and structural sub-models, that constructs model hypotheses on the basis of theoretical research or empirical rules (Iacobucci, 2009). The SEM consists of measurement and structural sub-models, which are analyzed separately below. The measurement and structural sub-models are described next, followed by the complete structural equation model.

#### Measurement Sub-Model

**FIGURE 1:**
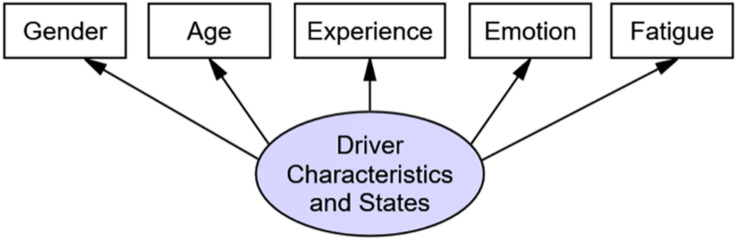
Measurement sub-model for driver characteristics and states variable as example.

The purpose of a measurement sub-model is to describe how latent variables are measured or conceptualized by the corresponding observed variables. The measurement sub-model in our study consisted of five latent variables: *road characteristics*, *driver characteristics and states*, *distraction elements*, *cognitive abilities*, and *SA*. Each latent variable was represented by several measurable, observed variables, as shown in Table 1. The variables of r*oad characteristics* were traffic volume, traffic complexity, and road complexity. The variables of *driver characteristics and states* were gender, age, driving experience, emotional state, and fatigue state. The *cognitive ability* variables were visual processing skills, working memory capacity, spatial perceptual ability, and time-sharing ability, based on the findings in recent research (O’Hare, 1997; Bolstad, 2001; Johannsdottir and Herdman, 2010). Based on the distracted driving behavior questionnaire (Zhang, 2018), the *distracting elements* included mobile phone use, conversation, eating, in-vehicle devices, absent-mindedness, and conditions outside the vehicle. Finally, based on the questionnaires relevant to drivers’ *SA* (e.g., Bolstad, 2001; Ma and Kaber, 2005; Kass et al., 2007; Jackson et al., 2009; Kaber et al., 2016; Taylor et al., 2016; Chandrasekaran et al., 2019), the observed variables for SA were 13 variables related to the level of perception (four variables), understanding (six variables), and prediction (three variables). Figure 1 shows an example of the measurement sub-model for the variable driver characteristics and states.

**TABLE 1:** Hypothesis of latent variables.

**Latent variables**		**Observed variables**
Road characteristics		Traffic volume (R1), traffic complexity (R2), road complexity (R3)
Driver characteristics		Gender, age, driving experience
Driver states		Emotional state (S1), state of fatigue (S2)
Distracting elements		Mobile phone use (D1), conversation (D2), eating (D3), in-vehicle devices (D4), absent-mindedness (D5), conditions outside the vehicle (D6)
Driver cognitive abilities		Visual processing skills (C1), working memory capacity (C2), spatial perceptual ability (C3), time-sharing ability (C4)
SA	Perception	Vehicles or pedestrians (SA11), traffic signs (SA12), speeds (SA13), perceived hazards (SA14)
	Understanding	Location/speed of vehicles around (SA21), sign content (SA22), sign line meaning (SA23), driving time (SA24), speed limit value (SA25), road name (SA26)
	Prediction	Safe overtaking/lane change/acceleration (SA31), driving behavior of surrounding vehicles/pedestrians at the next moment (SA32), predicting driving time (SA33)

#### Structural Sub-Model

**FIGURE 2:**
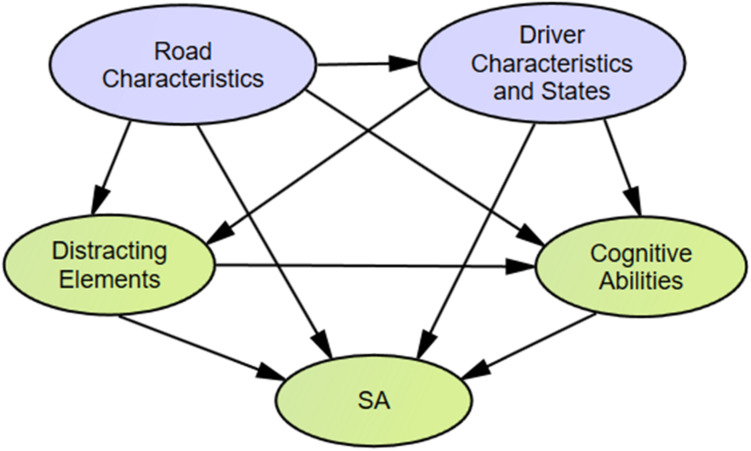
Hypothesis of structural sub-model.

The main purpose of a structural sub-model is to describe the relationship between the latent variables. When considering the road characteristics that drivers encounter, it can be assumed that driver characteristics and states, distracting elements, and cognitive ability will impact it and vice versa. That is, the road characteristics have an impact on other factors, and SA is affected by other factors. Given this, our main hypotheses are shown in Figure 2, as follows:

H1: Road characteristics impact driver characteristics and states, distracting elements, cognitive abilities, and SA.

H2: Driver characteristics and states can affect distracting elements, cognitive abilities, and SA.

H3: Distracting elements can affect cognitive abilities and SA.

H4: Cognitive abilities impact SA.

#### Combined Measurement and Structural Sub-Models

**FIGURE 3:**
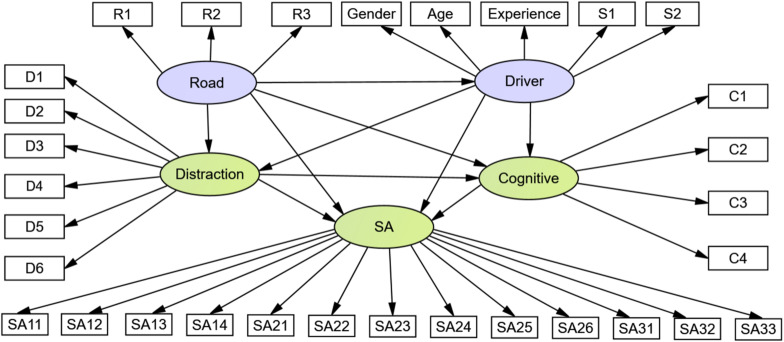
Path of structural equation model.

According to the hypothesis of the measurement and structure sub-model, the SEM path diagram of influential factors of drivers’ SA can be obtained, as shown in Figure 3. As gender, age, and driving experience in driver characteristics belong to classification variables, the internal consistency of driver characteristics is low. In the following analysis, the three variables were extracted, and the paths of the three variables were drawn, respectively. Meanwhile, the variables of driver state are emotion and fatigue.

## Methods

### Questionnaire Design

Based on the hypothesis of the measurement sub-model, the scale indices previously mentioned, and related theories, a questionnaire was developed to measure SA and its influential factors (see Table 1). As the scale was used to validate SEM, it was decided that a seven-point Likert scale would be implemented (Bollen, 1989). The three driver characteristic variables were represented as follows: gender (1 for male, 0 for female), age (1 for youth, 2 for middle-aged, 3 for elderly), and driving experience (1 for novice, 2 for general, 3 for experienced). The remaining observation variables were divided into seven levels (with 1 representing strong disagreement and 7 representing strong agreement). Before the formal survey, 50 pre-survey questionnaires were collected and reviewed, and the questionnaire was adjusted and revised based on the pre-survey results (Wang et al., 2019). The final formal questionnaire contained 31 questions. The composition and key points of the questionnaire are listed below in Table 1.

### Data Collection

To ensure the validity of collected questionnaire data, we conducted a survey of drivers who had driving experience in the 3 days prior to completing the survey. A total of 341 questionnaires were distributed, and 324 valid questionnaires were collected. The rate of valid questionnaires received was 95%. The information distribution of the samples is shown below in Table 2.

**TABLE 2:** Results of descriptive statistics.

**Characteristics**	**Classification**	**Sample**	**Percentage**
Gender	Male	228	70.4%
	Female	96	29.6%
Age	Youth (18–35 years)	266	82.1%
	Middle-aged (36–50 years)	52	16.0%
	Elderly (51–60 years)	6	1.9%
Driving years	≤6 years	258	79.6%
	>6 years	66	20.4%
Driving kilometers	≤50,000 km	255	78.7%
	>50,000 km	69	21.3%

## Results

Table 3 contains the means, standard deviations, and Pearson correlations among the variables. SA was positively correlated with age (*r* = 0.172, *p* < 0.01), experience (*r* = 0.197, *p* < 0.01), and cognitive ability (*r* = 0.46, *p* < 0.01), while it was negatively correlated with road characteristics (*r* = −0.269, *p* < 0.01) and distraction (*r* = −0.193, *p* < 0.01).

**TABLE 3:** Mean, standard deviation, and Pearson correlation coefficient (*N* = 324).

**Variable**	***M***	***SD***	**1**	**2**	**3**	**4**	**5**	**6**	**7**	**8**	**9**
1. Gender	0.70	0.46	1								
2. Age	29.27	7.96	0.14**	1							
3. Experience	5.01	5.36	0.11*	0.30**	1						
4. Emotion	4.69	1.02	0.10	0.08	0.06	1					
5. Fatigue	4.79	1.07	0.05	0.15*	–0.03	0.62**	1				
6. Cognitive	5.05	0.87	0.14*	0.36**	0.32**	0.15**	0.14*	1			
7. Environment	4.04	0.99	–0.03	–0.03	–0.03	–0.04	–0.04	–0.09	1		
8. Distraction	2.94	0.95	0.08	–0.08	−0.16**	–0.02	0.04	−0.13*	−0.12*	1	
9. SA	4.73	0.82	0.06	0.18**	0.20**	–0.09	–0.08	0.46**	−0.27**	−0.19**	1

*p < 0.05; **p < 0.01. SA, situation awareness.

### Reliability and Validity Analysis

Reliability tests, including Cronbach’s αcoefficient and composition reliability (CR) are generally used to check the consistency or stability of measurement data in analyses of questionnaire data. Each potential variable corresponds to a questionnaire. The Cronbach’s α and CR are calculated by SPSS 24.0 and AMOS 22.0, respectively, as shown in Table 4. The Cronbach’s α and CR of all questionnaires were greater than 0.7, which indicated acceptable reliability as well as high reliability and good internal consistency of the questionnaire (Hassan and Abdel-Aty, 2011; Wang et al., 2019).

**TABLE 4:** Results of tests of reliability and validity.

**Latent variables**	**Cronbach’s α**	**CR**	**AVE**
Road characteristics	0.738	0.754	0.529
Driver states	0.764	0.775	0.639
Distracting elements	0.841	0.858	0.503
Cognitive abilities	0.885	0.902	0.696
SA	0.933	0.950	0.593

*CR, composition reliability; AVE, average variance extracted.*

Validity tests are used to determine whether selected measurement variables are truly representative of the latent variable in question, as well as whether the latent variable can be completely and accurately measured using those latent variables (Martynova et al., 2018). Convergence validity is generally judged by average variance extracted (AVE) values; the higher the AVE, the higher the reliability and convergence validity of latent variables. AVE greater than 0.5 is the ideal standard value (Fornell and Larcker, 1981). As shown in Table 4, the AVE values of all latent variables in this study were greater than 0.5, indicating that the questionnaire had good convergence validity and was suitable for SEM analysis.

### Model Verification

#### Performance of Different Models

The consistency between our hypothesis model and survey data was judged by a model-fitting index. The Chi-square (χ^2^) and its significance level, the ratio of the Chi-square and degrees of freedom (χ^2^/df), the root-mean-square error of approximation (RMSEA), the goodness of fit index (GFI), the adjusted GFI (AGFI), and the comparative fit index (CFI) were collectively used as the evaluation index of model fitting. The model was analyzed via AMOS software. The fitting results are shown in Table 5. The GFI, AGFI, and CFI values of Model 1 before revision did not reach the recommended values, and so the model needed to be revised.

**TABLE 5:** Model-fit indices.

**Fit index**		**χ^2^**	***p***	**χ^2^/df**	**RMSEA**	**GFI**	**AGFI**	**CFI**
Before modified	Model 1	668.456	0.001	1.603	0.043	0.866	0.841	0.637
After modified	Model 2	493.481	0.001	1.272	0.029	0.901	0.874	0.848
	Model 3	494.989	0.001	1.269	0.029	0.901	0.874	0.848
Recommended value		—	—	(1,3)	<0.05	>0.90	>0.90	>0.90

*RMSEA, root mean square error of approximation; GFI, goodness of fit index; AGFI, goodness of fit index; CFI, comparative fit index.*

**FIGURE 4:**
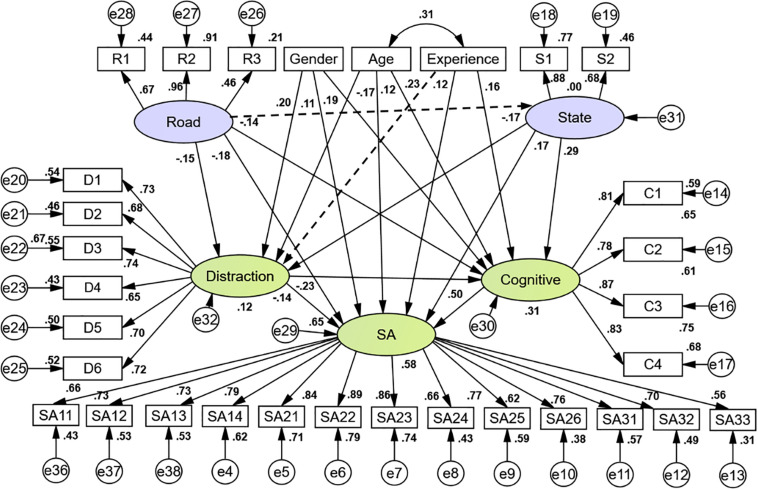
Drivers’ SA influential factors path relationship.

To achieve reasonable logic within the model, the path with a larger Modification Index (MI) value in the output of SEM could be added to realize model modification. To conform to the model logic, according to the MI of Model 1, the corresponding path was increased from large to small, and the modified Model 2 was obtained. The fitting results are shown in Table 5, which indicate that the fitting degree of Model 2 is acceptable (Zhang et al., 2018; Wang et al., 2019). The drivers’ SA influential factors path relationship is shown in Figure 4 for Models 1 and 2. Note that the two models include all relationship arrows (solid and dashed).

#### Verifying Model Hypotheses

The validity of a model hypothesis can be tested via the *p*-value of its path coefficient. The generalized least squares method was used to estimate the SEM. As shown in Table 6, only two paths were not significant: road characteristics to drivers’ state and driving experience to distraction. For this reason, these two paths were removed from Model 2 (dashed lines in Figure 4) to form Model 3. The path coefficient between the other latent variables was significant at the level of 0.05, which indicated that most of the model hypotheses were supported.

**TABLE 6:** Estimation results.

**Path**	**Model 1**		**Model 2**		**Model 3**	
	**Coefficient**	***P*-value**	**Coefficient**	***P*-value**	**Coefficient**	***P*-value**
Road → State	0.074	0.359	0.099	0.223	—	—
Road → Distraction	–0.146	0.04	–0.148	0.039	–0.15	0.033
Gender → Distraction	0.211	0.003	0.205	0.004	0.204	0.004
Age → Distraction	–0.168	0.019	–0.17	0.018	–0.171	0.012
Experience → Distraction	0.01	0.893	0.003	0.970	—	—
State → Distraction	–0.174	0.014	–0.163	0.022	–0.174	0.015
Road → Cognitive	–0.139	0.034	–0.137	0.042	–0.141	0.035
Gender → Cognitive	0.197	0.001	0.190	0.002	0.189	0.002
Age → Cognitive	0.217	0.001	0.226	0.001	0.226	0.001
Experience → Cognitive	0.17	0.006	0.16	0.012	0.157	0.013
State → Cognitive	0.294	0.001	0.284	0.001	0.290	0.001
Distraction → Cognitive	–0.228	0.001	–0.235	0.001	–0.234	0.001
Road → SA	–0.163	0.008	–0.175	0.005	–0.181	0.004
Gender → SA	0.115	0.036	0.112	0.041	0.113	0.036
Age → SA	0.117	0.031	0.12	0.028	0.121	0.026
Experience → SA	0.117	0.021	0.122	0.018	0.12	0.018
State → SA	0.15	0.019	0.169	0.009	0.171	0.008
Distraction → SA	–0.135	0.027	–0.133	0.030	–0.136	0.024
Cognitive → SA	0.509	0.001	0.507	0.001	0.500	0.001

## Discussion

Model parameter results are shown in Table 6 and Figure 4, which show standardized load and path coefficients. The load coefficients in the measurement sub-model indicate the extent to which the observed variables reflect the information of the latent variables. As shown in Figure 4, the load coefficients in this model were almost all greater than 0.6, which indicates that these observed variables could well reflect their corresponding latent variables. The path coefficients in the structural sub-model indicate the degree of influence among the latent variables. The total effect was the sum of the direct and the indirect effects. According to Table 6 and Figure 4, the effects of various factors on a driver’s SA are calculated, as shown in Table 7.

**TABLE 7:** Effects of various factors on SA.

**Effect**	**Road characteristics**	**Gender**	**Age**	**Experience**	**Driver states**	**Distracting elements**	**Cognitive abilities**
Direct effect	−0.181	0.113	0.121	0.120	0.171	−0.136	0.500
Indirect effect	−0.033	0.043	0.156	0.078	0.189	−0.117	—
Total effect	−0.213	0.156	0.277	0.198	0.360	−0.253	0.500

All factors had significant effects on SA. The positive effects were: cognitive abilities (0.500), drivers’ state (0.360), age (0.277), driving experience (0.198), and gender (0.158). The negative effects were: distraction (−0.253) and road characteristics (−0.213). Among all factors, drivers’ cognitive ability had the greatest influence on SA and was significantly higher than all other factors. This indicates that cognitive ability could be the dominant factor in the formation and maintenance of SA. Once cognitive ability is disturbed, SA is also adversely affected. The cognitive abilities affecting SA are mainly visual processing skills, working memory capacity, spatial perceptual ability, and time-sharing ability (Bolstad, 2001; Kaber et al., 2012). It is noted from Figure 4 that the four factors have similar reflections on cognitive abilities. This indicates that in complex traffic environments, drivers need to constantly mobilize their visual processing skills, working memory capacity, spatial perceptual ability, and time-sharing ability in order to maintain a good level of SA. However, drivers’ cognitive ability is affected by numerous factors. Other factors that affect SA could also affect cognitive abilities, most notably age, driver state, and distraction.

Drivers’ state had the second-highest level of influence on SA, which indicated that a positive emotional and mental state could help to form good SA. On the contrary, if drivers’ emotional and mental states were negative, anger, fatigue, or other negative emotions would diminish their accurate interpretation of the road environment and/or vehicle state and would ultimately result in distraction and slow movements.

Among driver characteristics, age had a positive impact on SA. As shown in Table 2, elderly drivers in the sample only accounted for 1.9%, meaning that the respondents were mainly young and middle-aged drivers. This indicated that the SA of middle-aged drivers was higher than that of young drivers, which is consistent with previous research (e.g., Jackson et al., 2009; Underwood et al., 2013; Crundall, 2016). Gender had a positive impact on SA, indicating that male drivers tended to have higher SA than female drivers.

Distraction emerged as having the greatest negative impact on SA. With increased frequency of driver distraction, SA gradually decreased. As distraction took up the drivers’ limited cognitive and attention resources, their SA was ultimately decreased due to their being engaged in other tasks and not paying attention to surrounding traffic. The strongest sources of distraction were mobile phone use and eating. Mobile phone use occupies a large amount of visual attention resources, while eating leads to greater visual-operational distraction. The factors that impacted distraction to a lesser degree were conversation and in-vehicle devices that tended to deplete drivers’ cognitive resources.

The negative impact of road characteristics on SA was obvious. The higher the road environment complexity was, the lower drivers’ SA was. When drivers’ attention was focused only on the surrounding vehicles and pedestrians, they could respond to emergencies in a timely fashion. However, distraction greatly reduced their perception of and reaction time to danger. This created conditions that were more likely to lead to accidents. Therefore, the negative impact of road characteristics on SA was smaller than that of distraction. From the load coefficient of the measurement sub-model, traffic complexity (e.g., vehicles/pedestrians crossing suddenly) better reflected road characteristics, followed by traffic volume and road complexity (e.g., presence of tunnels, bridges, and/or long downgrades).

## Conclusion

In this study, five latent variables (road characteristics, driver characteristics and states, distracting elements, cognitive abilities, and SA) and 31 observed variables were selected to establish a comprehensive structural equation model of the factors that influence a driver’s SA the most. All the preceding variables showed significant effects on SA. Specifically, the positive effects were (from large to small): cognitive abilities (0.500), drivers’ state (0.360), age (0.277), driving experience (0.198), and gender (0.156). Cognitive abilities played the most important role in improving driver’s SA, which is of great significance for drivers’ schools to carry out driver training. Driving schools should enhance their training to improve drivers’ cognitive abilities, including visual processing skills, working memory capacity, spatial perceptual ability, and time-sharing ability while driving. The negative effects were (from large to small): distraction (−0.253) and road characteristics (−0.213). The negative impact of distraction was greater than that of road characteristics, where both depleted drivers’ cognitive resources. Therefore, drivers should avoid distracted driving, which greatly reduces SA and can lead to serious traffic accidents. Stricter regulations prohibiting distracted driving should also be considered in traffic safety laws.

## Data Availability Statement

All datasets presented in this study are included in the article/Supplementary Material.

## Ethics Statement

Ethical review and approval was not required for the study on human participants in accordance with the local legislation and institutional requirements. The patients/participants provided their written informed consent to participate in this study. Written informed consent was obtained from the individual(s) for the publication of any potentially identifiable images or data included in this article.

## Author Contributions

YY, MC, CW, and XZ conceived the study. YY and MC contributed to investigation. YY, MC, and CW contributed to data curation. YY, MC, and SE contributed to writing paper drafts. YY and SE contributed to formal analysis. CW, SE, and XZ contributed to supervision. YY and XZ contributed to validation. All authors contributed to the article and approved the submitted version.

## Conflict of Interest

The authors declare that the research was conducted in the absence of any commercial or financial relationships that could be construed as a potential conflict of interest.

## Supplementary Material

FILE S1 The raw data of this study.

FILE S2 The questionnaire.
