# Supplementary material for: Structural Equation Modeling of Drivers’ Situation Awareness Considering Road and Driver Factors
Source: Front Psychol. 2020 Jul 21;11:1601. doi: 10.3389/fpsyg.2020.01601 (PMC7385403; doi:10.3389/fpsyg.2020.01601)
Supplement: FILE S2 — The questionnaire. [file Table_2.DOC]

**Appendix: Questionnaire used for data collection**

1. Have you driven a motor vehicle in the past 3 days?
2. *Yes B. No (If No, End of questionnaire)*
3. What is your gender?
4. *Male B.Female*
5. How old are you?
6. What is your driving age ? (/year)
7. How many kilometers have you driven? (/Ten thousand kilometers)

*A. 0-10 B. 10-30 C. 30-50 D. 50-100 E. 100-300 F. 300-600 G. 600-1000 H. ＞1000*

1. What was your emotional state during your last driving？
2. *Extremely negative B. Very negative C. Negative D. Neutral E. Positive F. Very positive G. Extremely positive*
3. What was your fatigue state during the last driving？

*A. Extremely fatigued B. Very fatigued C. Fatigued D. Neutral E. Energetic F. Very energetic G. Extremely energetic*

1. Please carefully read each the sentence below, and judge and select the option that best describes your situation. Options are 1 to 7 (1 = Strongly disagree, 7 = Strongly agree)

| (a) When you drive normally, you will not only look at the traffic conditions in front, but also frequently and quickly look at the left and right rear-view mirrors to pay attention to the traffic conditions in the rear and on the side. |
| --- |
| (b) When you drive, you often know when and where to look. |
| (c) You can clearly remember the traffic scene (including intersection / traffic sign information / vehicle color, etc.) |
| (d) You can quickly identify vehicles and pedestrians from a scene. |
| (e) You have a strong sense of time, such as knowing clearly how long it takes to complete a task. |

1. Please recall your latest driving situation. Read each sentence below carefully and select the option that best matches your situation. Options are 1 to 7 ( 1 = Never, 7 = Always )

| (a) How often did you use your phone while driving? (e.g. watching messages, sending messages, voice calls) |
| --- |
| (b) How often did you chat with passengers during the driving? |
| (c) How often did you eat (drink water and eat) while driving just now? |
| (d) How often did you regulate the on-board equipment during the driving process? (e.g. air conditioner, car navigation, car CD, radio) |
| (e) How often did you lose your mind while driving? |
| (f) How often did you look at the billboards on the side of the road during driving just now? |

1. Please answer your most recent driving situation. Read each sentence carefully to determine how well each description fits your situation and make your choice. Options are 1 to 7 (1 = Strongly disagree, 7 = Strongly agree).

| (a) The road you just drove is very congested. |
| --- |
| (b) The traffic conditions on the road you just traveled are very complicated (e.g. there are many pedestrians / electric vehicles crossing or retrograde) |
| (c) There are many accident-prone roads on the road you just traveled (e.g. long downgrades, sharp bends, intersections, tunnel entrances, highway entrances) |
| (d) During the driving process just now, you can always pay attention to the surrounding vehicles or pedestrians (e.g. vehicles coming to and behind, pedestrians walking crossing the road). |
| (e) You can always quickly notice every sign on the road and see the contents clearly. |
| (f) You can sense the speed of your vehicle correctly all the times, with an error of ± 5km / h. |
| (g) When there is a danger on the road (for example, a vehicle suddenly enters the lane), you can always notice the danger quickly. |
| (h) You can quickly determine the relative position, distance and speed of the surrounding vehicles. |
| (i) You can always quickly get and understand the information you need from the signboard. |
| (j) You can always correctly understand the meaning of all traffic markings and signs on the road. |
| (k) You can calculate exactly how long it takes you to cross a road section. |
| (l) You always know the speed limit on your road. |
| (m) You always know the name of your road. |
| (n) You can always quickly predict whether the current conditions can safely overtake, change lanes, accelerate or decelerate. |
| (o) You can always predict the driving behavior of the surrounding vehicles/pedestrians in the next moment (e.g. acceleration, deceleration, lane change, overtaking / moving forward, and turning) |
| (p) Whether the road is congested or smooth, you can always accurately predict the time when you arrive at your destination. |
